# Image-enabled cell sorting permits prospective isolation of primitive hematopoietic stem cell subsets with distinct functional potential

**DOI:** 10.1016/j.exphem.2026.105457

**Published:** 2026-08

**Authors:** Lilia Cabrera-Cosme, Ellie Bennett, Joanna Miłek, Zarema Albakova, Alexander J. Hogg, Sophie J. Davies, Sukhveer Kaur Mann, Karen Hogg, Peter O’Toole, David G. Kent

**Affiliations:** a Department of Biology, Centre for Blood Research, York Biomedical Research Institute, University of York, York, United Kingdom; b Department of Biology, Biosciences Technology Facility, University of York, York, United Kingdom

## Abstract

Fluorescence-activated cell sorting (FACS) has been a core technology for the identification, characterization and isolation of hematopoietic stem cells (HSCs), allowing for prospective isolation of cells which express specific molecules at specific levels of intensity. A recent technological breakthrough, image-enabled cell sorting (ICS), is based on FACS but it also permits separation of cells based on the differential distribution of these molecules across the cell. Using ICS in primary HSCs, we surveyed the patterns of expression for a range of intracellular markers and identified mitochondrial dyes as a tool to prospectively isolate HSCs with different properties. Integration of index-sort data with single-cell divisional kinetics identified total intensity and diffusivity of the MitoTracker dye as parameters enabling prospective isolation of functionally heterogeneous HSCs. *In vivo* functional assays confirmed that HSCs capable of long-term multilineage contribution were highly enriched in the MitoTracker low diffusivity, low intensity populations. Moreover, HSCs displaying high MitoTracker intensity but low MitoTracker diffusivity (Mito^±^), displayed the characteristics of low MitoTracker intensity (Mito^−/−^), representing a subpopulation of HSCs only isolatable by tools such as ICS. Collectively these data demonstrate that ICS is a powerful tool for prospectively enriching highly specific cell populations in the hematopoietic system, making it broadly useful for applications where population identification and purity are required to attain robust cellular and molecular characterization.

## HIGHLIGHTS

• Live imaging-enabled sorting strategy using MitoTracker Green-Fei Mao fractionates hematopoietic stem cells (HSCs).
• Single-cell in vitro assays show differences in quiescence exit between HSC subsets
• Serially reconstituting HSCs in the low total intensity, low diffusivity fraction

## Teaser

For decades, haematopoietic stem cells (HSC) have mainly been isolated by combinations of cell surface markers, which limits our understanding of the prevalent functional heterogeneity across the HSC pool. Here, we have leveraged a novel live-imaging enabled cell sorting (ICS) technology to isolate HSC subsets inferred from imaging and index-sort data. The combination of two imaging parameters, total intensity and diffusivity, made it possible to prospectively isolate and functionally characterise different MTG-FM HSC phenotypes. This work demonstrates the power of ICS to help tackle challenges in understanding HSC functional heterogeneity.

## Graphical abstract

**Figure:**
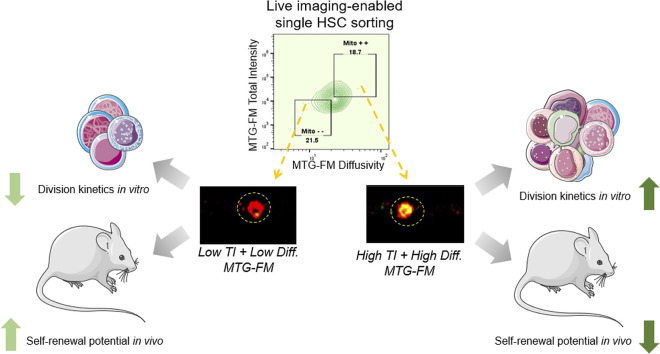

Prospective isolation of HSCs was catalyzed by two landmark technological inventions: fluorescence-activated cell sorting (FACS) [1, 2, 3] and monoclonal antibodies [4,5]. FACS in particular has allowed researchers to obtain and functionally assess highly enriched populations of rare cell populations in the blood and immune system and has led to their detailed cellular and molecular characterization, such as HSCs and B-cell populations [6]. Although numerous strategies for prospectively identifying highly purified HSCs have been published over the last decades [7, 8, 9, 10, 11], they are all uniformly reliant on FACS.

As a high-throughput multiparameter technology, FACS has limitations in the level of detail with which individual cells and the expression of molecules can be interrogated. Although the total amount of fluorescence of a specific marker is recorded, researchers are typically blind to the pattern of each marker's expression. Image-enabled cell sorting (ICS) [12] now permits these patterns to be observed in real time, allowing prospective isolation of cells with distinct patterns of expression. Recent applications utilizing this technology have yielded highly optimized platforms for doublet discrimination in cytometry [13] and approaches for identifying mislocalized proteins [14], but no studies to date have investigated its application in understanding HSC population heterogeneity. The ability to visualize the cell population of interest, in addition to spectral sorting, has recently further enabled the optimization of protocols to isolate cellular populations, for instance, mouse enteric populations [15].

The description of substantial heterogeneity in HSCs with respect to their self-renewal durability and the numbers and types of blood cells that they produce [10,16, 17, 18, 19, 20] makes them an ideal cell system to explore the utility of ICS. Although tools such as index-sorting have allowed for retrospective coupling of cellular and molecular function [21, 22, 23, 24], they do not allow researchers to directly link the activity of specific molecules with downstream in vitro and in vivo function. One such source of functional heterogeneity among the HSC pool has been attributed to the rate of metabolic activity in HSCs, particularly, mitochondrial activity. HSCs were initially described as possessing fewer mitochondria than actively cycling cells [25, 26, 27, 28, 29]. However, the introduction of reporter mice based on the photo-activatable mitochondrial (PhAM) system [30,31], has recently challenged the initial views on mitochondrial mass in HSCs [32,33]. Over the last decade, HSCs have been found to have elevated numbers of mitochondria [34, 35, 36], as well as increased Δψ_mt_ [37,38], collectively reshaping the view of mitochondrial activity in HSCs.

In this study, we utilized ICS to survey a range of intracellular markers in primary mouse HSCs. In particular, we focused on markers which report the activity and location of intracellular organelles to identify heterogeneity in these parameters that could be leveraged to improve prospective isolation of different HSC subtypes. Mitochondrial and lysosomal markers displayed considerable heterogeneity in their pattern of expression in HSCs and, using ICS, we isolated functionally distinct populations of mouse HSCs and assessed the function across in vitro and in vivo assays.

## METHODS

### Mice

All experimental animals were obtained from and housed at the Biological Services Facility of the Department of Biology, University of York. Mice were fed sterile food pellets and given water ad libitum. Experimental procedures were performed under project license numbers PEAD116C1 and PP9339623 granted to Professor Kent in accordance with the guidelines of the Animal Scientific Procedures Act 1986 and with the approval of the Animal Welfare and Ethical Review Body of the University of York. Wild-type C57BL/6J and B6.SJL-Ptprc^a^Pep^c^BoyJ (CD45.2) were used for HSC isolation, and c-Kit (CD117) deficient C57BL/6J/Ly5.1^w41/w41^ and B6.CD45.1 mice were used as recipients for transplantation experiments. CD45.1/CD45.2^+^ C57BL/6 mice were used to isolate supportive bone marrow cells.

### HSC Isolation and Organelle Staining

Bone marrow was harvested from femurs, tibiae, iliac bones, and spine from wild-type mice. Bones were crushed using a mortar and pestle, and subsequently passed through a 70-μm cell filter to create a single-cell suspension. Red blood cells were depleted by incubating in ammonium chloride solution (5 mL) for no more than 10 min, as previously described [39,40]. The cell suspension was enriched for hematopoietic stem and progenitor cells (HSPCs) by depleting mature cells using immunomagnetic selection via the EasySep mouse hematopoietic progenitor enrichment kit (StemCell Technologies, cat. no. 19856A). The HSPC-enriched suspension was stained at a 1:1000 concentration with the MitoTracker Green-Fei Mao (MTG-FM) [41] dye or the LysoTracker Green DND-26 (LTG) (Invitrogen) in phosphate-buffered saline (PBS), 1 × (Sigma–Aldrich, cat no. D8537-500ML) for 60 minutes at room temperature in the dark. Samples were centrifuged at 300*g* for 5 minutes to rinse off excess dye. For pharmacologic inhibition of Ca2+ pump experiments, cell suspensions were treated with verapamil hydrochloride (Sigma–Aldrich) (50 µM final concentration) or reserpine (Sigma–Aldrich) (25 µM final concentration), incubating for 30 minutes at room temperature. Following organelle staining, antibody staining was performed by addition of antibodies described in Supplementary Table E1. Samples were incubated with the antibody cocktail for 30 minutes on ice in the dark, washed and centrifuged to remove excess antibodies. Samples were filtered through a 40-μm cell strainer into PBS (1–2 mL) in preparation for cell sorting.

### Live Imaging-enabled Cell Sorting (ICS)

EPCR^+^CD45^+^CD150^+^CD48^-^ (ESLAM) HSCs were isolated from adult mouse bone marrow, having previously been shown by single-cell transplantation assays to be highly enriched (∼50%) for long-term reconstituting HSCs [10,39]. HSC subsets identified by live imaging parameters were analyzed and sorted using the BD FACSDiscover S8 instrument with BD FACSChorus software v. 6.2.0 (BD Biosciences), with a spectral array configuration consisting of five fluorescence lasers (UV, violet, blue, yellow-green and red). In addition, it consists of three photomultiplier tubes image-enabling blue-laser detectors [534/46BP (B1), 600/60BP (B2), and 788/225BP (B3)]. The B1 detector was selected to image organelles stained with MTG-FM or LTG; B3 was selected to detect expression of CD45 (BB700). Live imaging of sorted events was enabled by the BD CellView imaging discovery technology (BD Biosciences). Fully detailed laser configuration can be found in Supplementary Table E2 (generated with BD Research Cloud). For spectral unmixing, the system-aware spectral algorithm of the integrated BD SpectralFX technology (BD Biosciences) was applied to all sorted samples. Single-stain controls for spectral unmixing consisted of cells for the auto-fluorescence, fully stained, MTG-FM only, and live-dead controls; compensation beads (Biolegend) were used for the rest of the markers (Supplementary Table E1). The instrument was set to single-cell deposition mode for single-cell sorts, and to purity mode for bulk-cell assay sorts. Nozzle size was 85 μm for all sorts. Index-sort data and live-imaging data were recorded for all single-cell sorts.

### Single-Cell Division Kinetics Assays

Single ESLAM HSCs were sorted into round-bottom 96-well plates (Falcon), with each well containing StemSpan SFEM (StemCell Technologies, cat no. 09650), supplemented with 1% Penicillin/Streptomycin (Gibco), 1% L-glutamine (Gibco), 0.2% 2-mercaptoethanol (2-ME, Gibco), 300 ng/mL stem cell factor (SCF), and 20 ng/mL Interleukin 11 (IL-11) (Bio-Techne). Cells were incubated at 37°C, 5% CO_2_, and 90% relative humidity for 10 days. Cell counts were performed by observing the cells under an inverted microscope every 22–24 hours for a total of 4 days (96 hours) in the manner previously described [42]. Clone sizes were estimated as previously described [42, 43, 44] as very small (VS), small (S), medium (M), large (L), or very large (VL) on day 10 of culture, before flow cytometry analysis with counting beads to more accurately determine clone size in larger clones (See Results, Figure 2).

### Flow Cytometry Analysis of Single-cell In Vitro Assays

VS and small clones were pooled together as a single sample for flow cytometry analysis to acquire bulk phenotyping data; medium, large and VL clones were analyzed individually. Each clone was transferred into 96-well V-bottom polypropylene plates (Greiner BioOne), which were centrifuged for 5 minutes at 300*g* at room temperature to remove media supernatant. Samples were stained with a panel described in Supplementary Table E3. When antibody staining was completed, samples were resuspended into a 1:1000 (7-Aminoactinomycin D) 7-AAD solution in PBS, 1 × . Plates were analyzed on a CytoFLEX LX355 or LX375, using the CytExpert Software v.2.4.0.28 for data acquisition (Beckman Coulter).

### In Vivo Limiting Dilution Assays

For in vivo limiting dilution assays, fractionated ESLAM HSCs were isolated from wild-type CD45.2 donor mice and sorted as described above. Cells were resuspended into 300 μL of sterile PBS, 1 × . CD45.1^W41/W41^ mutant mice were used as primary transplantation recipients and were sublethally irradiated and immediately reconstituted with 5 or 20 fractionated ESLAM HSCs. Chimerism was monitored by serial bleeds performed every 4 weeks, for a total of 16 weeks. Blood samples were withdrawn via the tail vein; red blood cell lysis was performed as described previously [39], and samples were stained as described above, using the antibody panel presented on Supplementary Table E4. Blood samples were analyzed on a BD LSRFortessaX-20, using the FACSDiva software v 8.0.1 (BD Biosciences). At the end of the longitudinal follow-up period, mice were culled and bone marrow and spleen were harvested from recipients for end point analysis and for cryopreservation of tissue (bone marrow only) for secondary transplantations.

### Secondary Transplantations

Cryopreserved whole bone marrow suspensions were rapidly thawed, filtered, resuspended into PBS + 10% fetal bovine serum and centrifuged to remove freezing medium containing dimethyl sulfoxide (DMSO). Samples were centrifuged and filtered once more to remove any DMSO remnants and resuspended into sterile PBS, 1 × . Cell counts were performed on a Countess instrument (ThermoFisher) to assess viability of samples. A total of 1 × 10^7^ viable bone marrow cells were injected into lethally irradiated CD45.1 recipients. Chimerism was monitored as described above. At the 16-week transplant end point, bone marrow and spleen were collected for flow cytometry analysis.

### Data Analysis

Single-cell live-imaging enabled sorting images were directly extracted for gating strategies and for visualization purposes. For statistical analysis, Flow Cytometry Standard files (.fcs files) and index-sort files (.csv) were exported from the FACSChorus database. Imaging or CellView files (.cvw) were extracted with the BD CellView Image Extractor software v 2.0.5 and imported along with the.fcs and.csv files onto FlowJo v 10.10 (BD Biosciences). For image analysis, the CellView plugin (BD Biosciences) was applied to the population of interest, i.e., ESLAM cells and images were visualized on the image wall. Filter sets [forward scatter (FSC), side scatter (SSC), light loss, and imaging channel sets] and channel overlay sets were applied uniformly to all samples in each FlowJo workspace corresponding to independent experiments. Analysis of index-sort data only was performed using GraphPad Prism v 10.1.2 (GraphPad software LLC). Analysis of 10-day assay data, serial bleed, and end point tissue chimerism data was performed on FlowJo and GraphPad.

## Results

### ICS Resolves Mitochondrial and Lysosomal Heterogeneity in Primary Mouse HSCs

**Figure 1:**
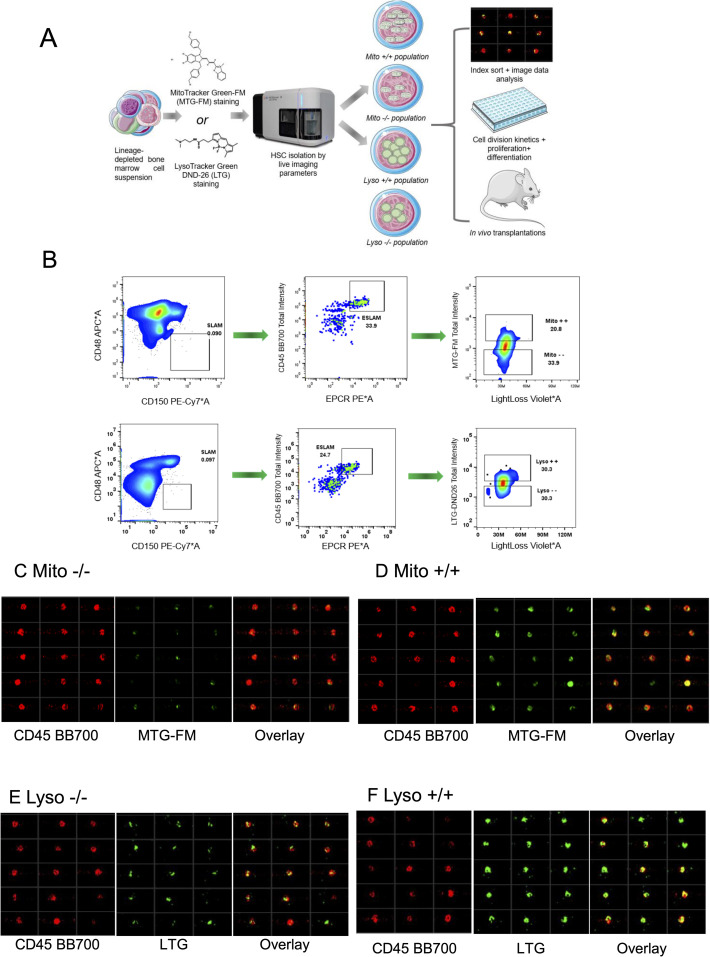
ICS of HSCs based on mitochondrial and lysosomal staining. **(A)** Experimental workflow to isolate primary mouse HSCs stained with MTG-FM or LTG-DND26. (**B)** Gating strategy for MTG-FM (top) and LTG-DND26 (bottom) stained cells. CD45 was used to corroborate viability of single cells by imaging. Following ESLAM HSC gating, TI of MTG-FM or LTG-DND26 was plotted against LightLoss (the parameter analogous to bright field of conventional microscopy images [12]). Cells were finally gated as high or low for the TI of each respective parameter. (**C–F)** Live-captured images of single HSCs sorted with CellView. The ROA and pixel threshold for each channel were adjusted before image acquisition and cell sorting. Images were acquired on the B1 and B3 image-enabling blue-laser detectors. A panel of ESLAM HSCs from representative experiments are shown where cells are gated as MitoTracker low **(C)**; MitoTracker high **(D)** LysoTracker low **(E)**; and LysoTracker high **(F)**. For each set of panels, images are displayed as CD45 BB700 (red, left), MTG-FM or LTG-DND26 (middle) and overlay of the two channels (right). Further representation of images processed through the CellView plugin for FlowJo can be found in Supplementary Figure E1.

To optimize experimental workflows, we performed single-cell sorts of primary mouse ESLAM HSCs [10,21,39] with additional gating for populations of high or low mitotracker green (MTG)-FM or LysoTracker green (LTG-DND26) in separate experiments. We first correlated total intensity (TI) of fluorescence with resulting clone size following 10 days of culture in supportive HSC conditions [43,45] to determine if there was any linkage to proliferative capacity. Next, using index-sort data collected from all of the additional imaging parameters [12], we retrospectively analyzed single sorted events using the FlowJo CellView plugin (Figure 1A).

For the first series of single-cell experiments, we selected TI (of MTG-FM or LTG). TI is derived from maximum intensity, which was originally described as an equivalent of the height (H) parameter in conventional flow cytometry [12]. However, maximum intensity takes the measure of all pixels in the image, inside and outside the region of analysis (ROA). TI, instead, provides the integration of all intensity for all pixels within the ROA, which gave a more accurate measure of the intensity of MTG-FM or LTG-FM, and allowed for more precise gating. Staining of CD45 surface expression ensured the appropriate selection of the ROA. As ICS does not permit real-time viewing of cells at speeds of >10,000 cells per second, gates for sort decisions must be made based on calculated numbers for each of the imaging parameters. To validate that sorting on calculated numbers successfully matched to images after the fact, ESLAM HSCs with high MTG-FM or LTG TI were sorted as per the gating strategy in Figure 1B. Visual confirmation of MTG-FM or LTG intensity was achieved using the B1(MTG-FM or LTG) and B3 (CD45 BB700) channels and a channel overlay (Figure 1C–F). Overall, these visual representations allowed discrimination of differences between cell populations that were high or low in MTG-FM or LTG intensity.

### Clonal In Vitro Assays Combined With Index-Sort Data Analysis Identify Two Key Novel Imaging Parameters to Classify HSCs

**Figure 2:**
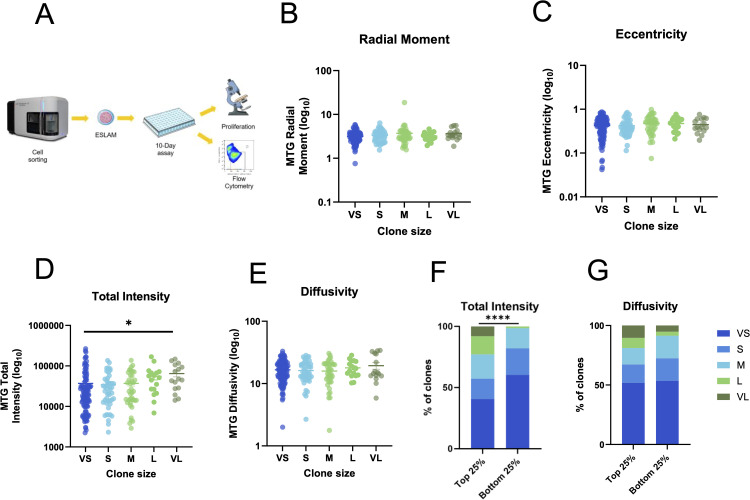
Single parameters of MTG TI or MTG diffusivity allow separation of HSCs with high proliferation capacity. (**A)** Single ESLAM HSCs were sorted on the basis of TI for MTG-FM and cultured in IL-11 and SCF whereupon clone size and phenotypic composition were analyzed. (**B–E)** Correlation of index-sort data of imaging parameters with clone size produced. Index-sort data were collected for each individual event sorted and categorized by clone sizes (VS, *n* = 116; S, *n* = 47; M, *n* = 39; l, *n* = 17; VL, *n* = 16). The MFI of radial moment (measure of the all-pixel distance relative to the centroid of the ROA) (B) and eccentricity (measure of the roundness of a marker, by dividing the long axis by the short axis of the ROA) remained uniform across all clone sizes. In contrast, a relationship between increase in clone size with an increase in MFI was found for TI (D), and a similar trend was observed for diffusivity (E). Most prominently, VL clones were the highest for TI of MTG-FM in relation to VS clones. VL and large clones displayed higher MFI of MTG-FM diffusivity than medium, small or VS clones. (F,G) Clone size distribution across the top (*n* = 58) and bottom (*n* = 58) 25% TI (F) and diffusivity (G) fractions. Cells in the top 25% fraction gave rise to a significantly higher proportion of L and VL clones (**χ**^2^, *p* < 0.0001), whereas cells in the top 25% fraction of diffusivity yielded an ∼twofold higher frequency of L and VL clones (**χ**^2^, *p* = 0.2251). Single HSCs found in the bottom 25% TI and diffusivity fractions gave rise to smaller clones, suggesting a new gating strategy based on live imaging parameters. ns = nonsignificant, *p < 0.05, ****p < 0.0001. *L* = Large; *M* = medium; *S* = small; *VL* = very large; *VS* = very small.

To determine the utility of the various imaging parameters available through ICS, we sorted single HSCs and cultured them for a total of 10 days in HSC maintenance conditions (300 ng/mL SCF and 20 ng/mL IL-11) [45] to assess proliferation and expression of mature cell markers (Figure 2A). Clone sizes were calculated using Precision Counting Beads (analogous to TruCount beads) as described previously [42], and these data were then correlated with index-sort data. Four imaging parameters were assessed (Figure 2) for their ability to discriminate HSCs with increased proliferation or differentiation—TI, diffusivity, eccentricity, and radial moment. Although we could not observe clear patterns using these parameters on the LysoTracker dye (Supplementary Figure E1), both the MTG-FM TI and the diffusivity imaging parameters appeared to separate HSCs with distinct proliferation capacity (Figure 2 B–G).

Diffusivity is a measure of the concentration of pixels corresponding to the intensity of a parameter’s signal within the ROA, allowing to discern the distribution of the signal of a given marker (here, MTG-FM) across the ROA. Radial moment and eccentricity are both metrics of particle shape [12]. Specifically, radial moment measures the spread of a particle’s signal calculated by the average distance of the pixels from the center of the ROA. Eccentricity is the ratio of a particle’s signal from the shortest to the longest axis within the ROA, giving out a measure of how elongated a particle will be. However, the radial moment and eccentricity parameters did not associate with clone size (Figure 2B,C), suggesting that these parameters did not significantly impact the proliferative capacity of HSCs in vitro. Importantly, there was a clear linear relationship between the MFI of TI of MTG-FM and the size of single-cell–derived clones at 10 days (Figure 2D). The MFI of MTG-FM diffusivity (which measures the concentration of the intensity of a given signal) also displayed a similar pattern, where clone size increased together with the MFI of diffusivity (Figure 2E). When separated into the top and bottom 25% total intensity and diffusivity, the proportion of large and VL clones was significantly higher using TI alone (*p* < 0.001, Figure 2F), and a similar trend was observed for diffusivity (Figure 2G). As these observations are based on retrospective index-sort data, we elected to take forward TI and diffusivity as parameters to prospectively isolate cells.

### Lysosomal Staining did not Resolve for Phenotypically Distinct HSC Fractions

Conversely, differences between the Lyso^−/−^ and Lyso^+/+^ phenotypes were not as consistent as for Mito^−/−^ and Mito^+/+^ (Supplementary Figure E2). Although the MFI of LTG-DND26 TI increased linearly with clone size (Supplementary Figure E2A), the MFI of diffusivity was largely comparable across all clone sizes, indicating that diffusivity did not resolve for HSC subsets as efficiently as it did for MTG-FM (Supplementary Figure E2B). Statistical testing of the proportion of clone sizes formed in vitro after 10 days of culture, there were no significant differences observed (Supplementary Figure E2C). Although a slight increase in VS clones produced by Lyso^−/−^ cells (52.33%) in relation to Lyso^+/+^ cells (37.38%) was observed, this was not sufficient to distinguish between the 2 HSC fractions.

To then characterize the kinetics of cell division of Lyso^-/-^ and Lyso^+/+^ HSCs, we carried out a 10-day clonal assay to count the number of divisions every 24 hours (Supplementary Figure E2E). Time-to-first division did not vary significantly between the Lyso^+/+^ and Lyso^−/−^ subsets, although a slight increase in the number of HSCs completing the second (or further) division across timepoints was observed (Supplementary Figure E2E). To obtain a precise quantification of cell number in Lyso^−/−^ and Lyso^+/+^ cultures, we performed flow cytometry analysis of cultures (Supplementary Figure E2D). As expected, absolute cell counts were similar between the two subsets. Phenotypically, cultures were likewise similar, as no evident differences were observed in frequencies of lineage-negative (Lin^−^, CD11b^−^ Ly6G/C^−^ (Gr-1)), Lin^-^ Sca-1^+^ c-Kit^+^ (LSK) or phenotypic HSC (EPCR^+^ CD45^+^ LSK) fractions (Supplementary Figure E1F).

Collectively, because these data indicated that ICS-enabled sorting of HSC fractions based on LTG-DND26 did not identify phenotypically defined subsets, this was not carried out into the next experimental phase.

### Prospective Isolation of HSC Subsets Inferred by the Integration of TI and Diffusivity of MTG-FM

**Figure 3:**
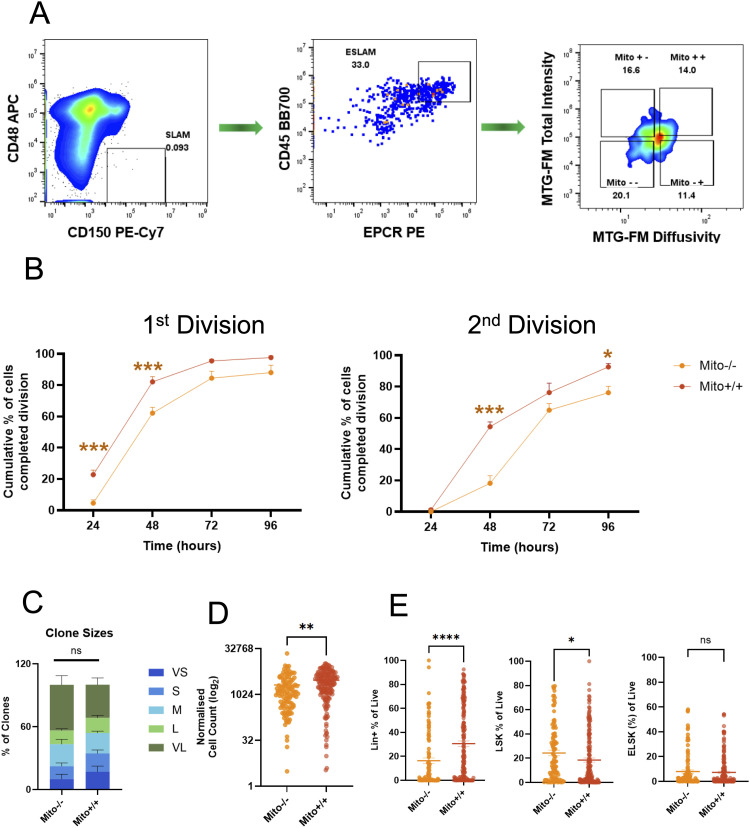
A novel ICS strategy for HSCs. (**A)** Subsequent to the traditional SLAM gate, EPCR was plotted against TI of CD45 BB700 (because this was used as a control for live image acquisition). ESLAM cells were then gated as: MTG-FM high total intensity, low diffusivity (Mito^±^); low TI, high diffusivity (Mito^-/+^); high TI, high diffusivity (Mito^+/+^); low TI, low diffusivity (Mito^−/−^). (**B)** Assessment of first (left panel) and second (right panel) cell cycle divisional kinetics of single Mito^−/−^ and Mito^+/+^ HSCs where a significantly higher proportion of Mito^+/+^ HSCs exited quiescence (*p* < 0.001) and underwent a second division at 48 hours (*p* < 0.0001) and 96 hours (*p* = 0.0094). (**C)** Categorical 10-day clone sizes generated by Mito^−/−^ and Mito^+/+^ HSCs (**χ**^2^, ns. (**D)** Normalized clone sizes at day 10 showing cell number is higher in cultures initiated with Mito^+/+^ HSCs (*p* = 0.0058). (**E)** Phenotypic composition of Mito^−/−^ and Mito^+/+^ clones showing reduced mature cell production (measured by Lin^+^, *p* < 0.0001) and increased HSPC production (measured by LSK, *p* = 0.359) in Mito^−/−^ cultures. ELSK proportions were not different between Mito^−/−^ and Mito^+/+^. ns = nonsignificant, * *p* < 0.05, ** *p* < 0.01, *** *p* < 0.001, **** *p* < 0.0001.

The systematic assessment of single-cell in vitro data coupled with index-sort data, led to the development of an HSC sorting strategy (Figure 3A). Because diffusivity is a nonconventional flow cytometry parameter derived from the maximum intensity of a single pixel within the ROA ^−^[12], real-time imaging data were crucial to inform the design of a novel gating strategy. The plotting of MTG-FM TI against diffusivity resulted in four different subsets of HSCs. For ease, these were labeled as Mito^+/+^ (high in both parameters), Mito^−/−^ (low in both parameters), Mito^+/−^ (high intensity, low diffusivity), and Mito^−/+^ (low intensity, high diffusivity). We first compared the 2 extremes Mito^+/+^ and Mito^−/−^ (Figure 3B), and observed that Mito^−/−^ cells exit quiescence more slowly (4.61% vs. 22.82% at 24 hours post sorting) and complete second division more slowly (18.24% vs. 54.39% at 48 hours post sorting).

These slower cell cycle kinetics also resulted in smaller clones being formed by Mito^−/−^ HSCs (Figure 3C,D) and the cells produced were on average more immature when compared with Mito^+/+^ cells (Figure 3E) as indicated by nearly twofold fewer Ly6G^+^ and CD11b^+^ cells and increased proportions of LSK cells. Because mitochondria play a significant role in calcium homeostasis in various cell types including HSCs [46], and this might result in efflux of dyes such as MTG-FM, we performed a control experiment using reserpine [47] and verapamil [48] (Supplementary Figure E3). Both compounds are calcium (Ca_2_) pump blockers and have previously been used in HSC purification methods [39,49]. Firstly, we did not observe major changes to the proportions in any of the HSC populations, except for a slight reduction of MTG-FM TI and diffusivity in both reserpine- and verapamil-treated samples (Supplementary Figure E3A). To confirm that we had isolated the appropriate phenotypes, index-sort data analysis confirmed the difference in TI and diffusivity between the Mito^−/−^ and Mito^+/+^ populations treated with reserpine (Supplementary Figure E3B) and verapamil (Supplementary Figure E3C). Ca_2_ pump blockage did not inflict major differences in clone size proportions (Supplementary Figure E3D) or the divisional kinetics (Supplementary Figure E3E,F) of Mito^−/−^ or Mito^+/+^ HSCs. Importantly, we noticed a decrease in the proportion of viable cells in the reserpine-treated sample, which is a common effect with this particular compound. Hence, we concluded that pharmacologic inhibition of Ca2+ pumps did not have a significant impact on the biology of the different phenotypes.

### ICS Permits Exclusion of Intermediate Phenotypes

**Figure 4:**
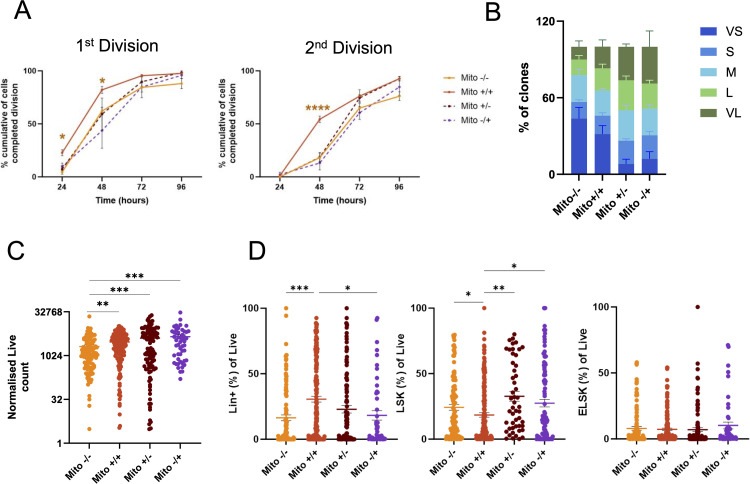
Intermediate phenotypes do not fully resemble Mito^+/+^ or Mito^−/−^ HSCs. (**A)** Divisional kinetics for both intermediate phenotypes (high intensity, low diffusivity [dotted maroon line] and low intensity, high diffusivity [dotted purple line]) compared with the Mito^+/+^ and Mito^−/−^ HSCs. Both intermediate phenotypes resemble kinetics of Mito^−/−^ HSCs, as these are slower in comparison to Mito^+/+^ HSCs (*p* = 0.05). (**B)** Categorical clone size assessment of all four HSC subsets where both Mito+/− and Mito−/+ gave rise to greater proportions of large and VL clones, outputs instead similar to Mito^+/+^ HSCs. **(C,D)** FACS analysis of single-cell in vitro assays of HSC subsets (Mito^−/−^*n* = 108; Mito^+/−^*n* = 104; Mito^−/+^*n* = 50; Mito^+/+^*n* = 173). (C) The calculated absolute number of cells in each clone revealed that Mito^−/−^ HSCs gave rise to the smallest clones overall, being significantly reduced compared with Mito^+/−^ (one-way ANOVA, *p* = 0.0010) and Mito^−/+^ (*p* = 0.0009), as well as Mito^+/+^ (*p* = 0.0058). (D) Phenotypic analysis of clone composition across subsets. (Left) The frequency of lineage-positive cells (calculated from total live cells) was significantly elevated in Mito^+/+^ HSC cultures in comparison to Mito^−/−^ (*p* = 0.0002) and Mito^−/+^ (*p* = 0.0301), but not Mito^+/−^ (*p* = 0.1144). (Center) Accordingly, a significant reduction in LSK frequency was observed in Mito^+/+^ HSC cultures in respect to all 3 other subsets (Mito^−/−^, *p* = 0.0359; Mito^+/−^, *p* = 0.0024; Mito^−/+^, *p* = 0.0194). (Right) The percentages of ELSK cells did not vary significantly between subsets (one-way ANOVA, *p* = ns). Significance symbols: * *p* < 0.05; ** *p* < 0.01; *** *p* < 0.001; **** *p* < 0.0001. *L* = Large; *M* = medium; *S* = small; *VL* = very large; *VS* = very small.

We next assessed cell divisional kinetics and clonal composition for the intermediate Mitotracker phenotypes (Mito^+/−^ and Mito^-/+^ HSCs) as it was unclear whether TI or diffusivity led to the functional differences described in Figure 3. When time-to-first and second division were measured (Figure 4A), both intermediate phenotypes resembled the Mito^−/−^ cell fraction, being significantly slower to enter division than the Mito^+/+^ cells. However, when the final 10-day clone size was assessed, both intermediate phenotypes gave rise to significantly larger clones than Mito^−/−^ cells, making them more similar to the phenotype of Mito^+/+^ cells (Figure 4B,C). Despite making increased numbers of cells per HSC, the proportion of lineage-positive cells was not increased in either of the intermediate cell populations compared with Mito^−/−^ cells and LSK retention was similar. Together, these data demonstrate that neither intermediate phenotype HSCs fully resemble the Mito^+/+^ or Mito^−/−^ HSCs, suggesting that the MTG-FM staining is a continuum rather than a tool to identify four discrete cell populations. A typical flow sort (e.g., without image-enabled acquisition) would prohibit separation of these four populations, leading to substantial functional heterogeneity that masks the phenotypes observed in Figure 3. Collectively, these data therefore highlight the utility of ICS for further purifying HSCs with distinct in vitro function.

To validate our MTG-FM–based gating strategy, we performed the same set of in vitro experiments with tetramethylrhodamine, ethyl ester, perchlorate (TMRE) to determine if we could phenocopy these results with a dye more specifically measuring mitochondrial membrane potential and observed similar changes in divisional kinetics but not in 10-day clone size or composition (Supplementary Figure E4). This suggests that although TMRE staining can partition HSCs into functionally distinct subsets, it was unable to resolve the same distinct subpopulations as MTG-FM pointing to mitochondrial membrane potential as less important for driving cell fate differences.

### ESLAM HSCs With Low MTG Staining are Enriched for Functional HSCs

**Figure 5:**
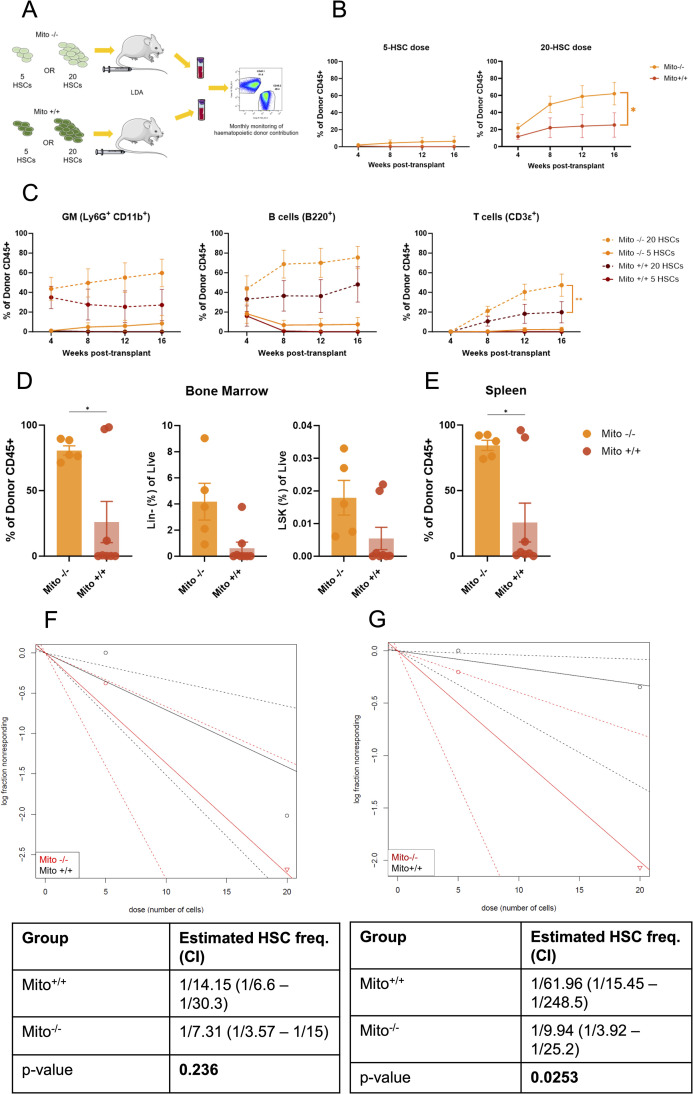
Mito^−/−^ HSCs have higher serial transplantation capacity. **(A)** Schematic of limiting dilution experiment. Limiting dilution assays (LDAs) were performed by sorting Mito^−/−^ and Mito^+/+^ HSCs over the course of two independent experiments. Two doses of HSCs from each subset were tested: 5 and 20 HSCs. A total of 32 recipients received HSC transfers (8 of Mito^−/−^ 5-HSC dose, 8 of Mito^−/−^ 20-HSC dose, 8 of Mito^+/+^ 5-HSC dose, and 8 of Mito^+/+^ 20-HSC dose). However, 1 recipient from the Mito^−/−^ 20-HSC group and 3 from the Mito^+/+^ 5-HSC group died due to causes unrelated to the transplant before the 16-week timepoint. Chimerism was monitored every 4 weeks for up to 16 weeks post-transplant by collecting peripheral blood (PB) from each recipient and analyzing by FACS. (**B)** Serial monitoring of PB showing donor chimerism over time. Although chimerism levels were low in 5-HSC dose recipients from both subsets, when transplanted with 20 HSCs, recipients of the Mito^−/−^ HSC fraction exhibited significantly higher donor contribution to overall blood cell production (two-way ANOVA, *p* = 0.0133). (**C)** Contribution to the granulocyte/monocyte (GM) (left), B-cell (center), and T-cell (right) lineages in each of the experimental groups across the time course, showing that all transplantations were multilineage. However, in both doses, the myeloid cell contribution was higher in the Mito^−/−^ cell fraction. The circulating T-cell proportion in Mito^−/−^ 20-HSC dose recipients was significantly elevated in comparison to Mito^+/+^ 20-HSC recipients (*p* = 0.0032). **(D)** Analysis of the donor-derived cell fraction in bone marrow (D) and spleen **(E)** of 20-HSC dose recipients. Overall chimerism in both bone marrow (D, left) and spleen (E) was increased by about threefold in the Mito^−/−^ recipients (*t* test, *p* = 0.0214 and *p* = 0.0111, respectively). In the bone marrow, the lineage-negative and LSK fractions (calculated from total live cells) were reduced in the Mito^+/+^ recipients (D, center and right). (F,G) ELDA test results for primary (F) and secondary (G) transplant experiments. Estimated HSC frequencies with confidence intervals (CI) are shown below each corresponding plot. In primary transplantation setting, the frequency of long-term self-renewing HSCs within the Mito^−/−^ fraction was estimated to be twice as higher than in the Mito^+/+^ fraction. The most striking difference was observed in the secondary transplant experiment, where the Mito^−/−^ fraction was estimated to contain six times more serially repopulating HSCs than Mito^+/+^ fraction. Importantly, all of the recipients within the Mito^−/−^ 20-HSC group were successfully repopulated, both in primary and secondary transplantation, denoted by the down-pointing red arrow on the bottom right corner of both ELDA plots. Two-way ANOVA (B,C) and *t* test (D), * *p* < 0.05, ** *p* < 0.01, *** *p* < 0.001, **** *p* < 0.0001.

To evaluate the in vivo functional capacity of the newly isolatable Mito^+/+^ and Mito^−/−^ cells, we performed limiting dilution transplantation assays using 5- or 20-HSC doses. Monitoring of chimerism showed similar levels of engraftment in early stages (Figure 5A) of the transplantation period but by 16 weeks, Mito^−/−^ cells displayed higher levels of overall chimerism compared with Mito^+/+^ cells (particularly at the 20 cell dose, *p* < 0.05, Figure 5B). Moreover, and consistent with previous descriptions [16] of myeloid chimerism tracking with greater HSC contribution, Mito^−/−^ cells had significantly increased relative contribution to myeloid cells at both doses (Figure 5C) and the also had higher levels of HSPCs and contribution to spleen hematopoiesis (Figure 5D). Upon secondary transplantation, these functional differences were further exacerbated, and the levels of calculated HSCs with secondary transplantation capacity were calculated using the Extreme Limiting Dilution Assay (ELDA) analysis tool on the Walter and Eliza Hall Institute (WEHI) website (https://bioinf.wehi.edu.au/software/elda/) [50] (Figure 5E,F). Strikingly, none (0%) of the 5-HSC Mito^+/+^ recipients were repopulated, whereas 3 of 8 (37.5%) 5-HSC Mito^−/−^ recipients showed >1% chimerism. Following the same trend, 75% of recipients (6/8) in the 20-HSC Mito^+/+^ group were repopulated compared with 100% of recipients (7/7) of the 20-HSC Mito^−/−^ group. Results of the ELDA analysis demonstrated a significantly increased frequency of reconstituting ESLAM cells (∼6-fold) in the Mito^−/−^ fraction when compared with the Mito^+/+^ fraction.

## Discussion

A growing body of literature points to several different causes for functional HSC heterogeneity, but methods to prospectively isolate functionally distinct HSCs hinder their delineation. In this study, using live-imaging-enabled cell sorting [12], we devised a novel gating strategy to functionally stratify HSC populations that is based on the imaging parameters of a mitochondrial marker, MTG-FM. We have defined our populations statistically with between 10% and 20% of HSCs falling within each quadrant, enabling isolation of functionally distinct subpopulations. For potential future experiments, multiple approaches might be pursued to improve separation of these functionally distinct populations including defining the populations biologically (e.g., a specific diffusivity threshold) or through use of mitochondrial reporter mice (e.g., mito-Dendra2 [30,34]).

Initial work has indicated that quiescent HSCs switch from glycolysis to oxidative phosphorylation (OXPHOS) upon cell cycle entry [51, 52, 53], and this would be accompanied by high mitochondrial activity. More recent studies support glycolytic metabolism in actively cycling HSCs [54, 55, 56], as well as alternative pathways such as the pentose phosphate pathway (PPP) [57,58]. This has led to the identification of high Δψ_mt_ in actively cycling HSCs [59,60], and together with mitochondrial content [36,61], Δψ_mt_ has equally been recognized to be heterogeneous in HSCs [38]. We found that Mito^+/+^ HSCs, high in MTG-FM TI and diffusivity (inferring higher Δψ_mt_), were more proliferative in vitro, exited quiescence more rapidly, gave rise to larger clones, and contained larger fractions of differentiated cells in culture. Our findings are similar to previous reports where low Δψ_mt_ was previously reported in ex vivo cultures as a marker of long-term reconstituting HSCs (LT-HSCs), as well as being associated with high expression of human HSC markers, including CD201 (EPCR), CD34, and CD90 [62].

In our transplantation experiments, donor Mito^−/−^ HSCs consistently repopulated primary and secondary recipients, clear evidence of retained functional potential [63, 64, 65]. Other studies have shown that low Δψ_mt_ measured by tetramethylrhodamine methyl ester (TMRM) was characteristic of repopulating murine HSCs [66]. Low Δψ_mt_ has also been reported as a feature of expanding HSPCs during development, which also regulates cell fate commitment [67], although the drivers of different metabolic requirements in adult and fetal HSCs remain unresolved.

The role of lysosomes in HSC quiescence has recently been described [55,68]. In our study, LTG-DND26 TI did not resolve for HSC heterogeneity. The affinity of LTG-DND26 for intracellular acidic compartments [55,69], may confound true staining of lysosomes with other acidic organelles [70,71]. Alternatives to LTG-DND26 or the development of reporter mouse strains might be more suitable in future for ICS-enabled sorting experiments focusing on lysosomal heterogeneity in HSCs.

One of the limitations of using MTG-FM is that it does not formally measure mitochondrial content in cells. This could be overcome by characterizing the imaging properties of HSCs isolated from the mito-Dendra2 reporter strain [31,36,72], although this may require evaluation of different imaging parameters to discern an association between mitochondrial content and functional HSC potential. Although the imaging capabilities of ICS are less powerful than microscopy tools such as confocal microscopy and should be utilized with caution, the thought that the distribution of mitochondria in an HSC might influence functional output is provocative. Potential areas of biological impact could be in the asymmetric partitioning of mitochondria in daughter cells [72,73], localized energy production [74], and/or calcium consumption [75] all leading to potential functional consequences for HSCs. Additionally, the visual assessment of the impact of reactive oxygen species (ROS) on the mitochondrial activity in HSCs [76] could help explain how this subsequently affects self-renewal potential. Expanding strategies that use ICS could have important implications for the identification of long-term reconstituting HSCs, for their ex vivo expansion [77] and use in the clinic [32].

## Declaration of competing interest

The authors do not have any conflicts of interest to declare in relation to this work.

## Appendix Supplementary materials

Image, application 1

Image, application 2

## Footnotes

Supplementary material associated with this article can be found in the online version at https://doi.org/10.1016/j.exphem.2026.105457.
