# Supplementary material for: Image-enabled cell sorting permits prospective isolation of primitive hematopoietic stem cell subsets with distinct functional potential
Source: Exp Hematol. 2026 Aug;160:None. doi: 10.1016/j.exphem.2026.105457 (PMC13446265; doi:10.1016/j.exphem.2026.105457)
Supplement: Supplementary file 1 [file mmc1.pdf]

Supplementary data

Supplementary Table 1 – Antibody panel for ESLAM HSC isolation

Marker	Fluorochrome	Clone	Concentration	Cat. no.	Supplier	Lot no.
CD45	BB700	30-F11	1:100	566439	BD Horizon	3150801
EPCR (CD201)	PE	eBio1560 (1560)	1:100	12-2012-82	Invitrogen	2600245
CD48	APC	HM48-1	1:100	103412	Biolegend	B341106
CD150	PE-Cy7	TC15-12F12.2	1:100	1155914	Biolegend	B353358
Sca-1 (Ly6A/E)	BV421	D7	1:200	108128	Biolegend	B419089
Fixable viability dye	R780	--	1:3000	65-0865-14	Invitrogen	2633408

Supplementary Table 2 – BD S8 Cytometer configuration (attached as PDF file)

Supplementary Table 3 – Antibody panel for *in vitro* assay 10-day timepoint analysis

**Marker**	**Fluorochrome**	**Clone**	**Concentration**	**Cat. no.**	**Supplier**	**Lot no.**
CD45	FITC	30-F11	1:200	103108	Biolegend	B330230
EPCR (CD201)	PE	eBio1560 (1560)	1:200	12-2012-82	Invitrogen	2600245
Gr-1 (Ly6C/Ly6G)	BV605	RB6-8C5	1:300	108439	Biolegend	B376581
Mac-1 (CD11b)	APC	M1/70	1:300	101212	Biolegend	B408151
Sca-1	BV421	D7	1:200	108128	Biolegend	B419089
c-Kit (CD117)	APC-Cy7	2B8	1:200	105826	Biolegend	B405703

Supplementary Table 4 – Antibody panel for transplant chimerism analysis

Marker	Fluorochrome	Clone	Concentration	Cat. no.	Supplier	Lot no.
CD45.1	PE-Cy7	A20	1:500	110729	Biolegend	B435520
CD45.2	AF700	104	1:500	109821	Biolegend	B375109
Ly6G	BV421	1A8	1:500	127628	Biolegend	B356918
CD11b	BV605	M1/70	1:500	101257	Biolegend	B407813
B220/CD45R	APC	RA3-6B2	1:500	103212	Biolegend	B379792
CD3e	PE	145-2C11	1:500	100308	Biolegend	B346465

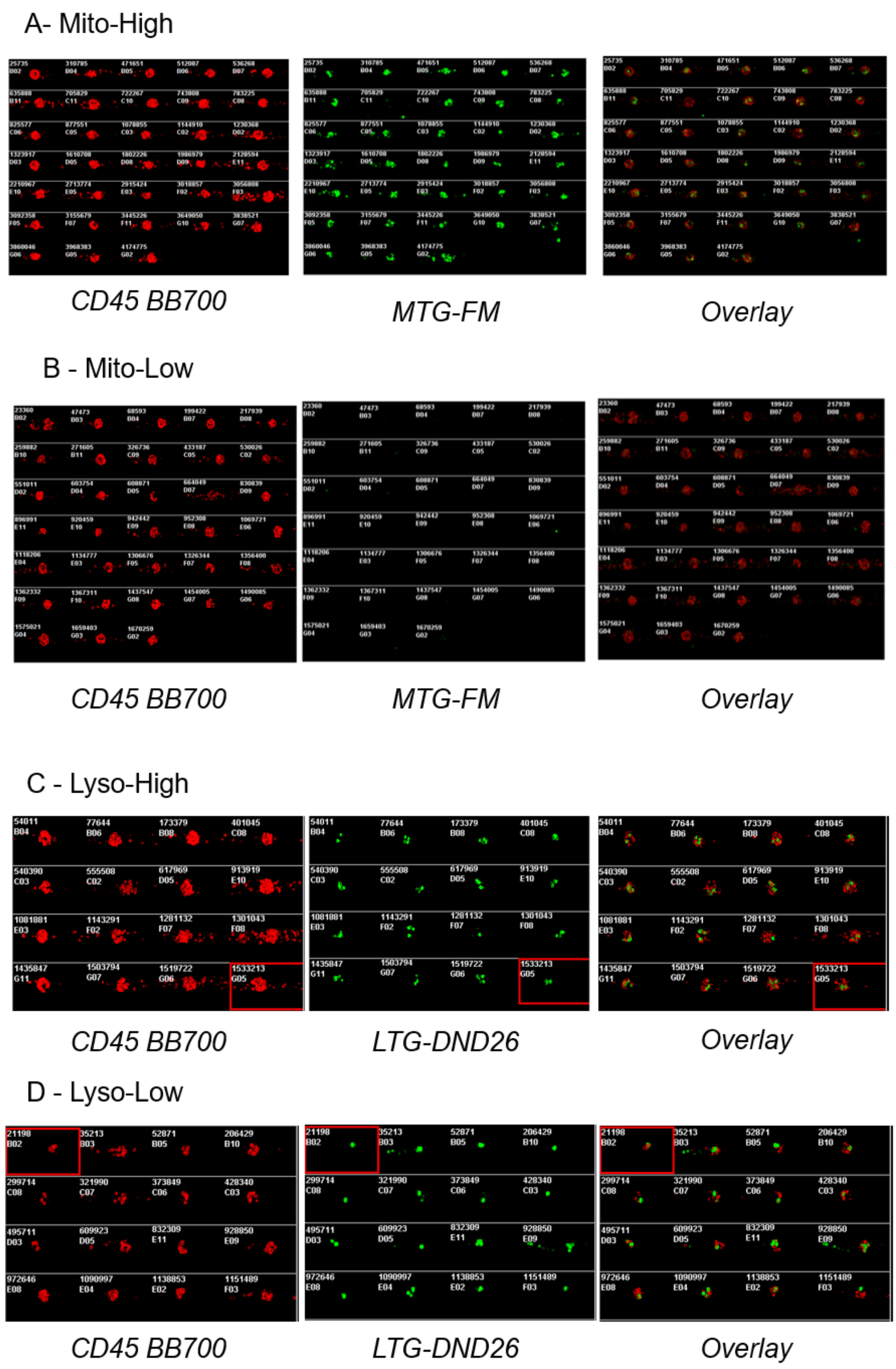

Supplementary Figure 1 (related to Figure 1) - Analysis of live-acquired image data of single HSCs sorted on the BD S8 with CellView for Flowjo. HSCs were stained with MTG-FM or LTG-DND26 and sorted as A) High MTG-FM TI; B) Low MTG-FM TI; C) High LTG-DND26 TI; D) Low LTG-DND26 TI. Images were collected from the ESLAM gate while performing an imaging-enabled cell sorting experiment. Data were exported from the BD S8 FACS Chorus software and processed using the CellView plugin (https://www.flowjo.com/exchange). Left panels show CD45 BB700 staining in red, middle panels show MTG-FM or LTG-DND26 staining in green, and right panels show a solid overlay of the two channels for each individual HSC.

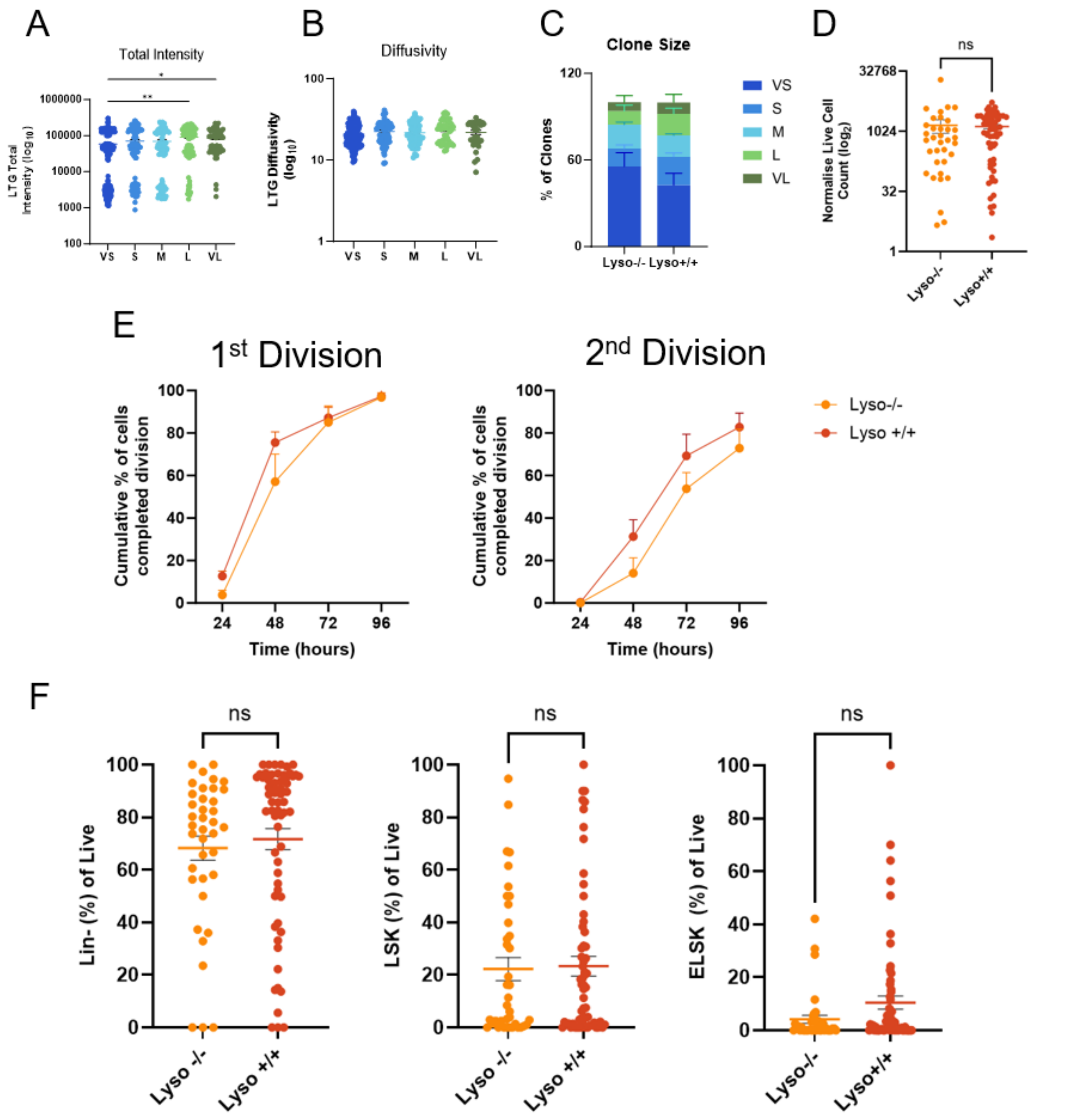

Supplementary Figure 2 (related to Figures 1 and 2) - Lyso$^{-/-}$ and Lyso$^{+/+}$ HSCs displayed similar division kinetics and phenotypic composition in culture. A-B) Index-sort data analysis of individual sorted HSCs categorised by clone sizes. A total of 600 cells were sorted (300 of Lyso$^{-/-}$ and 300 of Lyso$^{+/+}$) across three independent experiments. A) The MFI of LTG-DNDN26 TI was significantly reduced in cells which gave rise to very small clones in relation to those generating large and very large clones. B) MFI of LTG-DND26 Diffusivity remained comparable across all clone sizes. C) The proportion of each clone size category did not vary significantly between Lyso$^{-/-}$ (n = 191) and Lyso$^{+/+}$ (n = 193) HSCs (two-way ANOVA, p = ns). D) Calculated absolute number of cells within each individual clones at 10 days of culture was comparable between Lyso$^{-/-}$ (n = 38) and Lyso$^{+/+}$ (n = 61). Data for two independent experiments are shown. E) A slightly reduced proportion of Lyso$^{-/-}$ HSCs remained quiescent in culture (left) before entering the cell cycle, but underwent a similar number of cell divisions as Lyso+/+. F) FACS analysis of Lyso$^{-/-}$ and Lyso$^{+/+}$ clone phenotypic composition in culture. The frequency of lineage-positive, LSK and ELSK cells was calculated from total live cells. One-way ANOVA (A and B) Two-way ANOVA (E), *t* test (D and F), * $p < 0.05$, ** $p < 0.01$, *** $p < 0.001$, $p < 0.0001$.

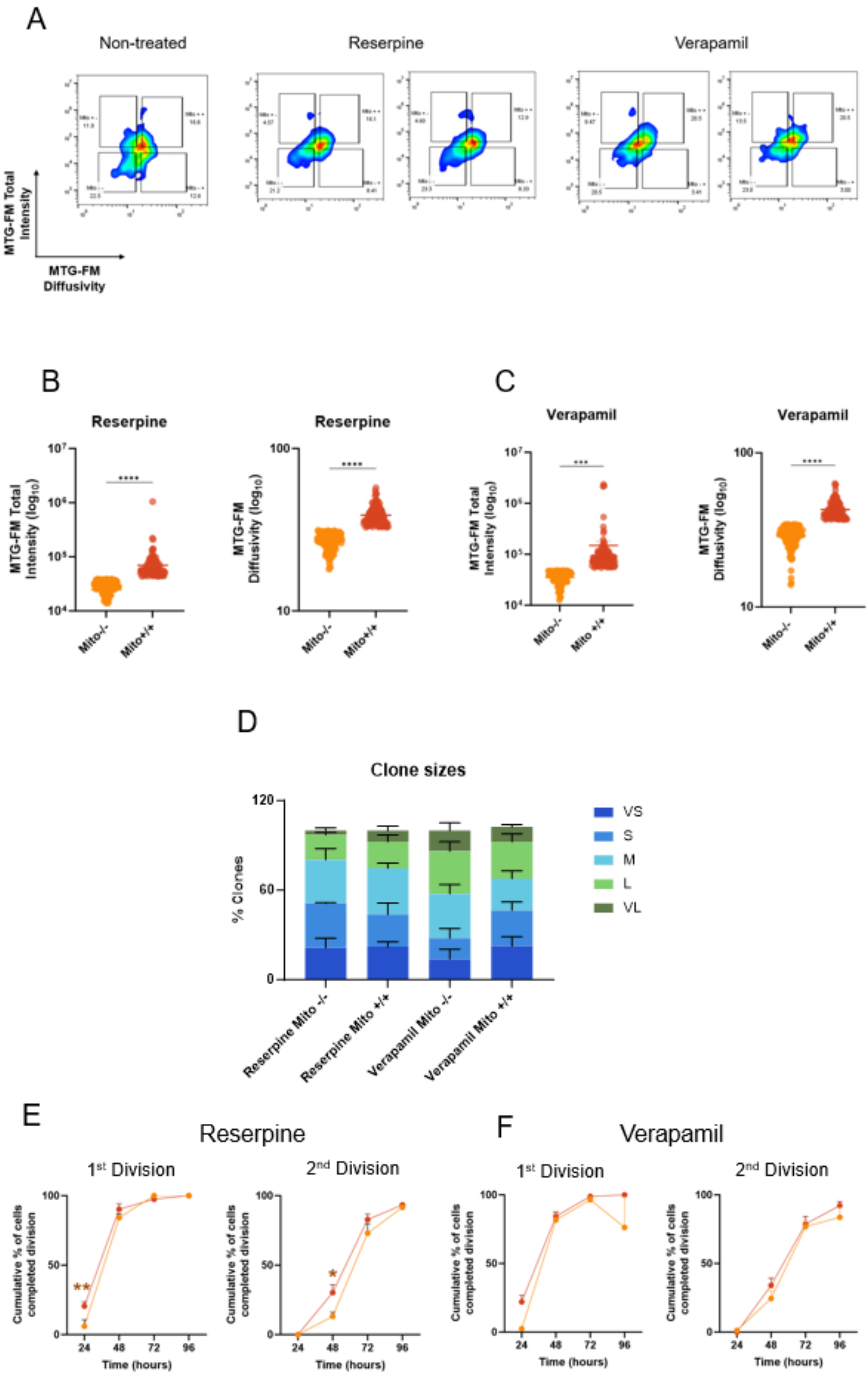

A
Non-treated
Reserpine
Verapamil
MTG-FM Total Intensity
MTG-FM Diffusivity
B
Reserpine
Reserpine
MTG-FM Total Intensity (log10)
MTG-FM Diffusivity (log10)
Mito-/-
Mito+/+
C
Verapamil
Verapamil
MTG-FM Total Intensity (log10)
MTG-FM Diffusivity (log10)
Mito-/-
Mito +/+
D
Clone sizes
% Clones
VS
S
M
L
VL
Reserpine Mito -/-
Reserpine Mito +/+
Verapamil Mito -/-
Verapamil Mito +/+
E
Reserpine
1st Division
2nd Division
Cumulative % of cells completed division
Time (hours)
F
Verapamil
1st Division
2nd Division
Cumulative % of cells completed division
Time (hours)

Supplementary Figure 3 - Pharmacological pump blocking with Verapamil and Reserpine of Mito-/- and Mito+/+ HSCs. A) Flow cytometric plots of Mito$^{-/-}$, Mito$^{+/+}$, Mito$^{+/-}$ and Mito$^{-/+}$ populations in non-treated, control (left), reserpine- (centre) and verapamil- (right) treated samples. A slight reduction in the TI and diffusivity of MTG-FM was observed as a result of pharmacological treatment. Analysis of cell viability (not shown) demonstrated a 20% reduction of viable cells in reserpine-treated samples. B-C) Index-sort data analysis of Reserpine (A) (n = 120) and Verapamil (B) (n = 120) treated lineage-depleted preparations for Mito$^{-/-}$ and Mito$^{+/+}$ single-HSC sorts. The MFI of MTG-FM TI (left) and Diffusivity (right) were assessed. The MFI for both TI and diffusivity in reserpine-treated cells was significantly higher in Mito$^{+/+}$ than in Mito$^{-/-}$ (p = 0.0032 and p = 0.0043. The same was observed for Verapamil blocking, where Mito$^{+/+}$ showed the highest TI and diffusivity (p <0.0001). D) Clone sizes remained at comparable proportions between the Mito-/- (reserpine-treated, n = 82; verapamil-treated, n = 78) and Mito$^{+/+}$ (reserpine-treated, n = 109; verapamil-treated, n = 76) fractions on either experimental condition (χ^2 =ns). E-F) Cell division kinetics of reserpine (E) and verapamil F) treated single HSCs. When treated with reserpine, Mito$^{+/+}$ HSCs were still faster to enter cell cycle (p = 0.0044), and underwent more divisions at the 48-hour timepoint only (p = 0.0138). Verapamil blocking did not impact exit of quiescence (left) or division kinetics (right) of Mito$^{-/-}$ or Mito$^{+/+}$ HSCs. * p < 0.05, ** p < 0.01, *** p <0.001, **** p < 0.0001.

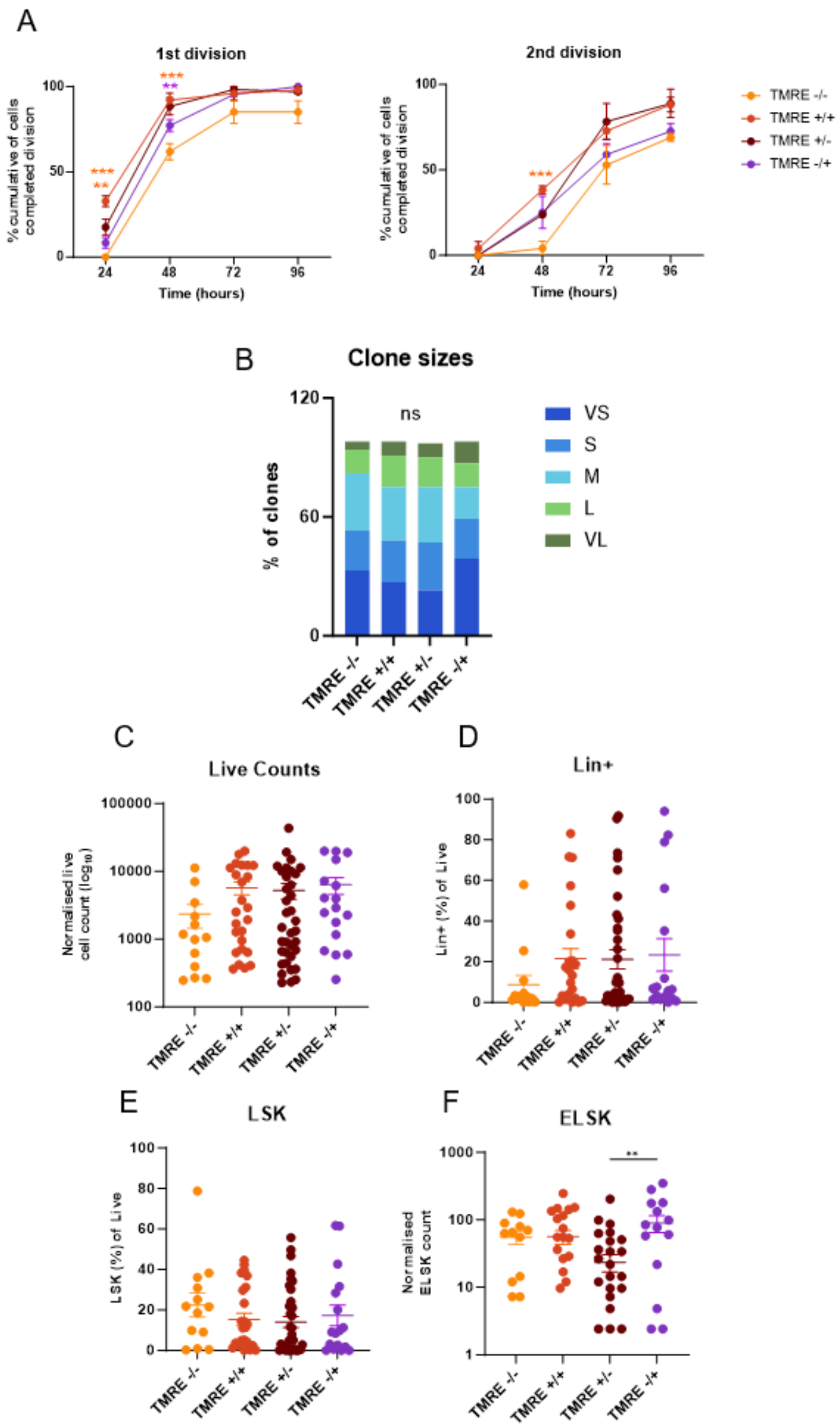

Supplementary Figure 4 – Prospective ICS-enabled isolation of HSC subsets by TMRE staining. A) Divisional kinetics of different HSC subsets. TMRE$^{+/+}$ HSCs enter the cell cycle significantly faster than TMRE$^{-/-}$ (p = 0.0003) and TMRE$^{-/+}$ (p = 0.0076) HSCs within 24 hours post-sorting (Reviewer Figure 1A). At 48 hours of culture, most of TMRE$^{+/+}$ and TMRE$^{+/-}$ cells had completed the first round of division in comparison to TMRE$^{-/-}$ cells (p = 0.0009 and p = 0.0034, respectively). In a similar fashion, a larger proportion of TMRE$^{+/+}$ HSCs underwent second division by 48 hours than in the TMRE$^{-/-}$ population (p = 0.0180) (two-way ANOVA). B) Clone size assessments of HSC subsets isolated by TMRE staining. We found no significant differences in the proportion of each of the clone sizes formed by clones of any of the phenotypes (χ^2 = 0.2975). C-F) Phenotypic analysis of 10-day single-cell cultures of HSC subsets. The number of viable cells (C) the frequencies and absolute numbers of haematopoietic stem and progenitor cells (D-E) did not differ significantly with an exception of elevated numbers of ELSKs in TMRE$^{-/+}$ cells relative to TMRE$^{+/-}$ (p = 0.0041) (one-way ANOVA). ** p<0.01; *** p<0.001.
