# Supplementary material for: Image-enabled cell sorting permits prospective isolation of primitive hematopoietic stem cell subsets with distinct functional potential
Source: Exp Hematol. 2026 Aug;160:None. doi: 10.1016/j.exphem.2026.105457 (PMC13446265; doi:10.1016/j.exphem.2026.105457)
Supplement: Supplementary file 2 [file mmc2.pdf]

Optical configuration

Introduction

The BD FACSDiscover™ S8 cell sorter is available in the following optical configurations:

Configuration	Lasers
5 laser (BRVYGUV)	Blue (B), Red (R), Violet (V), Yellow Green (YG), and Ultraviolet (UV)
4 laser (BRVYG)	Blue (B), Red (R), Violet (V), and Yellow Green (YG)
4 laser (BRVUV)	Blue (B), Red (R), Violet (V), and Ultraviolet (UV)
3 laser (BRV)	Blue (B), Red (R), and Violet (V)

Optical filter layout

The term "detector" refers to any of the three different types of detectors on the instrument: photodiode (PD), photomultiplier tube (PMT), or avalanche photodiode (APD). See Optical specifications (page 424).

The following table shows the optical filter layout for the 5 laser configuration. For the other laser configurations, the optical filter layout for the lasers in the respective configuration remains the same.

Laser	Detector name	Band pass filter
Blue	B1 (500)	501/16
	B2 (515)	517/16
	B3 (530)	532/16
	B4 (545)	547/16
	B5 (575)	574/16
	B6 (590)	590/16
	B7 (605)	607/18
	B8 (625)	625/18
	B9 (655)	655/20
	B10 (675)	675/20
	B11 (700)	698/26
	B12 (725)	724/26
	B13 (750)	752/30
	B14 (780)	782/30
	B15 (810)	812/30
	B16 (845)	844/34
Imaging	FSC	N/A
	SSC (Imaging)	488/15
	Light Loss (Imaging)	488/5
	ImgB1 (535)	534/46
	ImgB2 (600)	600/60
	ImgB3 (790)	788/225
Red	R1 (655)	655/20
	R2 (675)	675/20
	R3 (700)	698/26
	R4 (725)	724/26
	R5 (750)	752/30
	R6 (780)	782/30
	R7 (810)	812/30
	R8 (845)	844/34

Laser	Detector name	Band pass filter
Ultraviolet	UV1 (375)	375/20
	UV2 (390)	392/14
	UV3 (420)	420/20
	UV4 (440)	440/20
	UV5 (460)	459/18
	UV6 (475)	476/16
	UV7 (500)	501/16
	UV8 (515)	517/16
	UV9 (530)	532/16
	UV10 (545)	547/16
	UV11 (575)	574/16
	UV12 (590)	590/16
	UV13 (605)	607/18
	UV14 (625)	625/18
	UV15 (655)	655/20
	UV16 (675)	675/20
	UV17 (700)	698/26
	UV18 (725)	724/26
	UV19 (750)	752/30
	UV20 (780)	782/30
	UV21 (810)	812/30
	UV22 (845)	844/34

Laser	Detector name	Band pass filter
Violet	SSC (Violet)	405/15
	Light Loss (Violet)	405/5
	V1 (420)	420/20
	V2 (440)	440/20
	V3 (460)	459/18
	V4 (475)	476/16
	V5 (500)	501/16
	V6 (515)	517/16
	V7 (530)	532/16
	V8 (545)	547/16
	V9 (575)	574/16
	V10 (590)	590/16
	V11 (605)	607/18
	V12 (625)	625/18
	V13 (655)	655/20
	V14 (675)	675/20
	V15 (700)	698/26
	V16 (725)	724/26
	V17 (750)	752/30
	V18 (780)	782/30
	V19 (810)	812/30
	V20 (845)	844/34

Laser	Detector name	Band pass filter
Yellow Green	YG1 (575)	574/16
	YG2 (590)	590/16
	YG3 (605)	607/18
	YG4 (625)	625/18
	YG5 (655)	655/20
	YG6 (675)	675/20
	YG7 (700)	698/26
	YG8 (725)	724/26
	YG9 (750)	752/30
	YG10 (780)	782/30
	YG11 (810)	812/30
	YG12 (845)	844/34
